# Bivariate genome-wide association study (GWAS) of body mass index and blood pressure phenotypes in northern Chinese twins

**DOI:** 10.1371/journal.pone.0246436

**Published:** 2021-02-04

**Authors:** Zhaoying Li, Weijing Wang, Xiaocao Tian, Haiping Duan, Chunsheng Xu, Dongfeng Zhang

**Affiliations:** 1 Department of Epidemiology and Health Statistics, the College of Public Health of Qingdao University, Qingdao, Shandong Province, People’s Republic of China; 2 Qingdao Municipal Center for Disease Control and Prevention, Qingdao, Shandong Province, People’s Republic of China; 3 Qingdao Institute of Preventive Medicine, Qingdao, Shandong Province, People’s Republic of China; Huazhong Agriculture University, CHINA

## Abstract

Recently, new loci related to body mass index (BMI) or blood pressure (BP) have been identified respectively in genome-wide association studies (GWAS). However, limited studies focused on jointly associated genetic variance between systolic pressure (SBP), diastolic pressure (DBP) and BMI. Therefore, a bivariate twin study was performed to explore the genetic variants associated with BMI-SBP, BMI-DBP and SBP-DBP. A total of 380 twin pairs (137 dizygotic pairs and 243 monozygotic pairs) recruited from Qingdao Twin Registry system were used to access the genetic correlations (0.2108 for BMI-SBP, 0.2345 for BMI-DBP, and 0.6942 for SBP-DBP, respectively) by bivariate Cholesky decomposition model. Bivariate GWAS in 137 dizygotic pairs nominated 27 single identified 27 quantitative trait nucleotides (QTNs) for BMI and SBP, 27 QTNs for BMI and DBP, and 25 QTNs for SBP and DBP with the suggestive *P*-value threshold of 1×10^−5^. After imputation, we found eight SNPs, one for both BMI-SBP and SBP-DBP, and eight for SBP-DBP, exceed significant statistic level. Expression quantitative trait loci analysis identified rs4794029 as new significant eQTL in tissues related to BMI and SBP. Also, we found 6 new significant eQTLs (rs4400367, rs10113750, rs11776003, rs3739327, rs55978930, and rs4794029) in tissues were related to SBP and DBP. Gene-based analysis identified nominally associated genes (*P* < 0.05) with BMI-SBP, BMI-DBP, and SBP-DBP, respectively, such as *PHOSPHO1*, *GNGT2*, *KEAP1*, and *S1PR5*. In the pathway analysis, we found some pathways associated with BMI-SBP, BMI-DBP and SBP-DBP, such as prion diseases, IL5 pathway, cyclin E associated events during G1/S transition, TGF beta signaling pathway, G βγ signaling through PI3Kγ, prolactin receptor signaling etc. These findings may enrich the results of genetic variants related to BMI and BP traits, and provide some evidences to future study the pathogenesis of hypertension and obesity in the northern Chinese population.

## Introduction

Hypertension and obesity are public health challenges worldwide [1, 2]. Hypertension increases the risk of several diseases, such as chronic renal disease, stroke, dementia and coronary artery disease, etc. [3–7]. Obesity is related to cardiovascular disease, diabetes, cancer and increase global burden worldwide [8–10]. Both of hypertension and obesity are complex chronic diseases effected by genetic and environmental factors [11, 12]. SBP, DBP and BMI are important anthropometric measurements of hypertension and obesity, respectively. A systemic review reported the estimated heritability of SBP ranging from 17% to 52%, and DBP from 19% to 41% [13]. Another review estimated the heritability of BMI from 47% to 90% [14]. There were some mechanisms linking obesity with hypertension, such as sympathetic nervous system activation, hypothalamic-pituitary axis dysregulation, insulin resistance/hyperinsulinemia, and renin-angiotensin-aldosterone system activation [15, 16]. Several twin studies previously presented multivariate modeling of BMI and BP to explore their common genetic and environmental background [17, 18]. And it was demonstrated that the genetic correlation between BMI and BP ranged from 0.15 to 0.49, and additive genetic factors accounted for 65%-86% of the phenotypic correlations between BMI and BP components [19–23].

Genome-wide association studies (GWAS) are used to nominate loci for complex diseases [24]. Recently, new loci related to BMI or BP have been identified in GWAS [25–27]. However, all associated loci can only explain 2.7% of total variance of BMI, 2–3% of SBP, and 1.3–2.6% of DBP [28, 29]. Therefore, more causal loci need to be found. Univariate GWAS has been conducted with single trait that failed to take into account clinical relatedness and correlation among phenotypes. Multivariate GWAS has higher power outperforming univariate approach to explore more single nucleotide polymorphisms (SNPs) and pleiotropic genes potentially related to correlated phenotypes [30, 31]. A key advantage of it is to demonstrate how genetic variants effect on correlated phenotypes directly, instead of coincidental overlap of two traits. Several bivariate analyses of SBP and DBP demonstrated some SNPs located in chromosomes 3 and 9 [32]. Also, some pathways were found associated with BMI and BP [29, 33]. However, limited bivariate GWAS about BMI and BP phenotypes were conducted in the northern Chinese population. And genetic variance and genes potentially related to hypertension and obesity still remain unknown, and thus need further exploration. Therefore, we designed this bivariate twin study to: (1) identify genetic variants associated with BMI-SBP, BMI-DBP and SBP-DBP, respectively; and (2) identify potential causal genetic variation; (3) determine the common pathways associated with hypertension and obesity.

## Methods

### Twin samples

The participants were recruited from Qingdao twin registry system by internet, newspaper and local community hospital. More details of recruitment have described in literatures [34, 35]. Questionnaire data and examination data were collected from all twin samples. The participants were included if: they were 18 years old and over; questionnaire data, examination data and laboratory data were available; and twin pairs can be followed up. The participants were excluded if: they were in pregnant or in lactation; with serious illness or cognitive impairment; taking anti-hypertensive or lose-weight medicine within one month, as well as the incomplete twin pairs. 380 pairs of eligible twin samples (243 pairs of monozygotic (MZ) twin and 137 dizygotic (DZ) twins) were included. Zygosity was identified by sex, blood type and DNA markers at Qingdao Blood Center. This study has been approved by the Regional Ethics Committee of the Qingdao CDC Institutional Review Boards.

### Phenotypes

BMI was defined as an individual’s body weight in kilograms divided by the square of his/her height in meters (kg/m^2^). Blood pressure was taken by a standard procedure using mercurial table stand model sphygmomanometer. Systolic blood pressure was defined as Korotkoff of phase I (appearance of sound), and diastolic blood pressure was defined as Korotkoff phase V (disappearance of sound). Average values were used after three measurement.

### Genotyping, quality control, and imputation

137 DZ pairs was genotyped using Infinium Omni2.5Exome-8v1.2 BeadChip (Illumina, SanDiego, California, USA) on autosomes. Ultimately, 1,338,796 SNPs from 274 samples were analyzed in this bivariate GWAS according to inclusion criteria (Calling rate > 0.98; minor allele frequency, MAF > 0.05; Hardy-Weinberg, Hwe significance > 1×10^−4^; locus missing <0.05) [20].

To identify new risk variants, IMPUTE 2 [36] software was performed to impute typed SNPs using 1,000 Genomes Project Phase 3 as the reference panel (105 Han Chinese South samples, and 103 Han Chinese Beijing samples) [37] according to Linkage Disequilibrium (*r*^*2*^ >0.6), MAF>0.05, and Hwe>1×10^−4^. Finally, 7,401,356 SNPs were included in this study.

### Statistical analysis

#### Heritability

Bivariate Cholesky decomposition model [38] was used to assess the genetic and environmental effects on phenotypic correlation between BMI and BP using Mx software package (http://www.vcu.edu/mx) with age and sex adjusted. Due to the deviation of the distribution, the Blom’s formula was used to guarantee the normal distribution or approximate normal distribution. Bivariate Cholesky model decomposed variations for both phenotype into additive genetic (A), common (shared) environmental (C), and unique /non-shared environmental (E) variance. Nested models were conducted by dropping A (CE model) or C (AE model) after fitting the full model (ACE). The likelihood ratio *χ2* tests compared whether the full model and nested model has significant difference [38, 39]. The parsimonious model was chosen according to lower Akaike’s Information Criterion (AIC) as the best fitting model [40].

#### Genotyping, GWAS and conditional analysis

The genome-wide efficient mixed-model association (GEMMA) [41] was applied Genetic relatedness matrix and Bayesian sparse linear mixed model [42] were performed to estimate associations between genetic variance and phenotypic pairs in SNP-based analysis of BMI-SBP, BMI-DBP and SBP-DBP for 137 DZ twins, with age and sex adjusted. The conventional Bonferroni-corrected threshold of *P* < 5 × 10^−8^ was adopted as significant threshold [43, 44], and the commonly used threshold of *P* < 1 × 10^−5^ as a suggestive evidence level [45, 46]. When none SNPs reaches significant threshold, suggestive evidence level was applied to find more SNPs. Quantile-Quantile plot (Q-Q plot) depicted and compared the distribution of observed *P-* values of SNPs against the expected *P-* values. The genomic inflation factor λ revealed if there was evidence of inflation of test statistics due to population stratification. Manhattan plots provided a compact visual way of demonstrating *P-* values of SNPs ordered by chromosome on the *x* axis. PLINK software was used to perform conditional analysis on GWAS data to identify independently associated variants [47]. The region containing top variant with ±5 Mb upstream and downstream was selected. And we re- evaluated each variant conditioning on the top variant. Then we repeated this process and tested whether still significant variants associated with phenotypic pairs. The conditional analysis consists of a new group of much less tests compared to the unconditional analysis, therefore, a separate *P*-value of 0.001 was adopted as significant threshold.

#### Expression quantitative trait loci (eQTL) analysis

Expression quantitative trait loci has been widely used to uncover effect of genetic variants on phenotypes [48, 49]. To determine the potential causal SNPs related to blood pressure and BMI, we queried relative SNPs and genes on Haploreg V4.1 (http://pubs.broadinstitute.org/mammals/haploreg/haploreg.php) (Accessed data: November, 2020; Last Update Date: 5th, November, 2015) [50] and publicly available eQTL data from Genotype-Tissue Expression (GTEx) Project (https://www.gtexportal.org/home/) (Accessed data: November, 2020; Latest Data Release: V8) [51]. *cis*-eQTLs were identified in adipose-subcutaneous, artery-aorta, artery-coronary, artery-tibial, brain-cerebellar-hemisphere, heart-atrial appendage, and heart-left ventricle tissue.

#### Gene-based analysis

We used VEGAS 2 [52, 53] to determine the association between specific genes and phenotypic pairs. All of the SNPs were from “1000G East ASIAN” sample. As we obtained 19001 genes, a *P-* value below 2.63×10^−6^ (0.05/19001) was considered to be genome-wide significant. *P-* value below 0.05 was considered as nominal significance threshold [54].

#### Pathway enrichment analysis

PASCAL programme was used to calculate pathway-scored [55]. The genetic markers SNPs were firstly mapped to genes, then pathway scores were calculated through combining corresponding gene scores. Pathways with high scores were selected and evaluated by chi-squared or empirical score. Pathways and corresponding annotation can be obtained from KEGG, Reactome, and Biocarta.

## Results

The characteristics of eligible participants are shown in Table 1. A total of 380 twin pairs are identified, including 137 dizygotic pairs. 380 twin pairs are used in heritability analysis. The mean age of participants is 51.5±7.6 years; the mean BMI is 24.3±3.3 kg/m^2^, and mean SBP and DBP are 130.8±20.1mmHg and 83.1±10.9mmHg, respectively. 137 DZ pairs were used in GWAS with mean age of 51.0±7.2 years, mean BMI of 24.6±3.1 kg/m^2^, and mean SBP and DBP of 132.5±20.2mmHg and 84.1±10.5mmHg, respectively.

**Table 1:** Characteristics of participants by sex.

	Variable	All		Female		Male	
		Mean±SD	N	Mean±SD	N	Mean±SD	N
**380 twin pairs**	**Age(year)**	51.5±7.6	760	50.9±6.7	392	52.1±8.5	368
	**BMI(kg/m**^**2**^**)**	24.3±3.3	760	24.3±3.4	392	24.3±3.1	368
	**SBP(mmHg)**	130.8±20.1	760	126.2±19.9	392	135.6±19.1	368
	**DBP(mmHg)**	83.1±10.9	760	80.2±9.9	392	86.1±11.0	368
**137 DZ pairs**	**Age(year)**	51.0±7.2	274	51.1±7.1	136	50.8±7.3	140
	**BMI(kg/m**^**2**^**)**	24.6±3.1	274	24.5±3.2	136	24.7±3.0	140
	**SBP(mmHg)**	132.5±20.2	274	129.6±20.7	136	135.3±19.4	140
	**DBP(mmHg)**	84.1±10.5	274	81.9±9.9	136	86.2±10.7	140

### Heritability analysis

**Fig 1:**
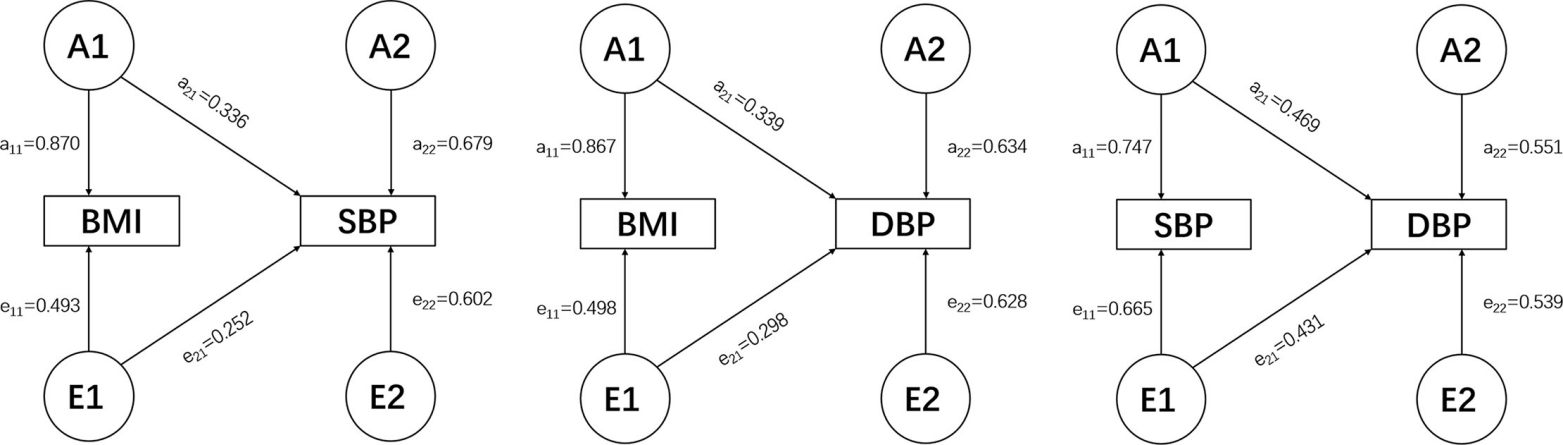
Best bivariate model (AE model) for BMI-SBP (A), BMI-DBP (B), and SBP-DBP (C) with standardized path coefficients. A1, A2: genetic variance components; E1, E2: unique environmental variance components; a11 through a22: genetic path coefficients, of which a22 demonstrates specific genetic influence on BP. e11 through e22: unique environmental path coefficients, of which e22 demonstrates specific unique environmental influence on BP.

The phenotypic correlation of BMI-SBP, BMI-DBP, and SBP-DBP are 0.214, 0.256, and 0.684, respectively under the best fitting model AE (S1 Table). Genetic correlations are 0.2108, 0.2345, and 0.6942, respectively (Table 2). The standardized path coefficients from best fitting AE model are presented in Fig 1.

**Table 2:** Estimated genetic and environmental correlations and phenotypic correlation between traits.

Phenotypic pairs	r_G_(95% CI)	r_C_(95% CI)	r_E_(95% CI)	Phenotypic correlation
**Full model**				
BMI-SBP	0.3299(-0.0694,0.7262)	-1(-1,1)	0.2302(0.1102,0.3437)	0.211
BMI-DBP	0.2082(-02678,0.6084)	-1(-1,1)	0.3218(0.2077,0.4274)	0.256
SBP-DBP	0.8604(0.3403,1)	0.3488(-1,1)	0.6627(0.5870,0.7268)	0.683
**Best fitting Model**				
BMI-SBP	0.2108(0.0843,0.3302)*	-	0.2360(0.1194,0.3462)*	0.214
BMI-DBP	0.2345(0.1027,0.3571)*	-	0.3183(0.2067,0.4218)*	0.256
SBP-DBP	0.6942(0.6006,0.7698)*	-	0.6728(0.6031,0.7323)*	0.684

r_G_: Genetic correlation; r_C_: Shared environmental correlation; r_E_: Non-shared environmental correlation.

∗: *p* < 0.05.

### Bivariate GWA analysis

#### SNP-based analysis, imputation and conditional analysis

**Fig 2:**
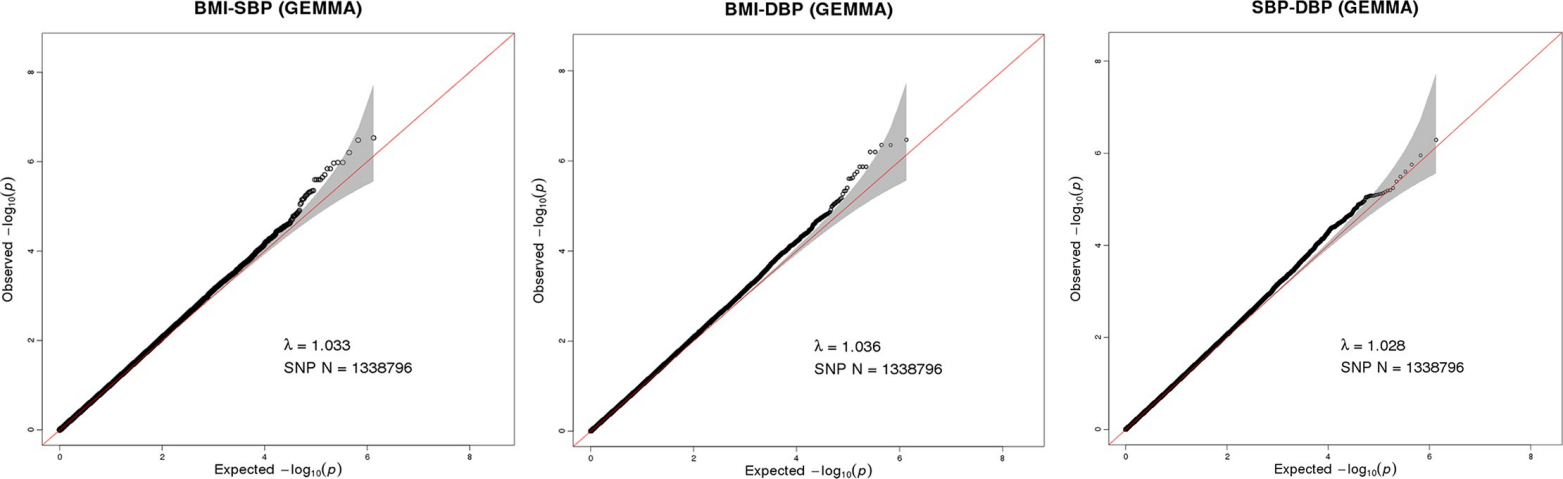
Q-Q plot for SNP associations with BMI-SBP (A), BMI-DBP (B), and SBP-DBP (C). The red line represents the expected value under the null distribution. The x- axis shows the expected -log10 *P*-values, while y-axis shows the observed -log10 *P*-values. The gray shaded gives the 95% acceptance region. N refers to the number of SNPs. λ refers to genomic inflation.

**Fig 3:**
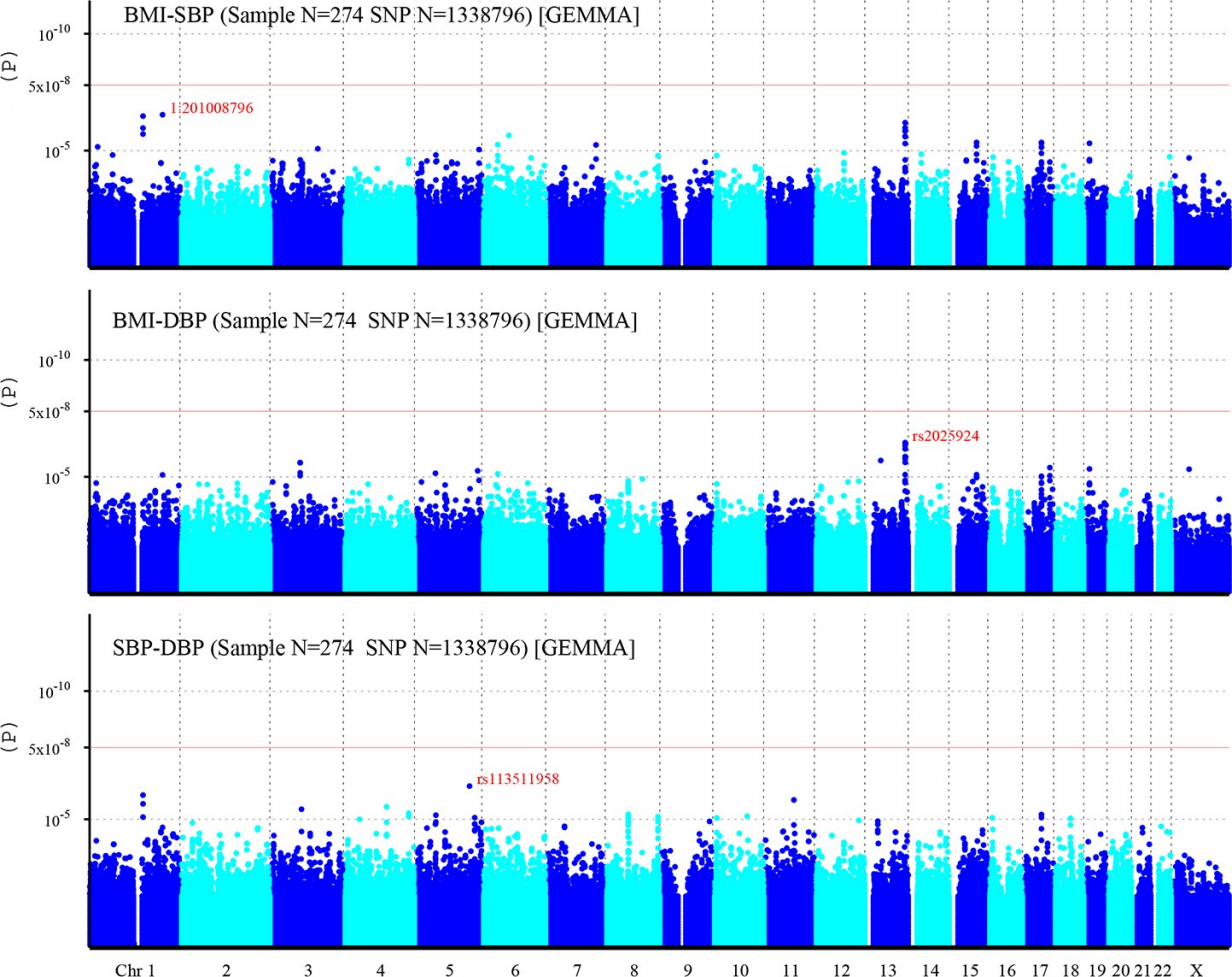
Manhattan plot of individual SNP associations with BMI-SBP, BMI-DBP, and SBP-DBP. Manhattan plot depicts the individual SNP ordered by chromosome. The x- axis shows the order of chromosome, while y-axis shows the *P*-values of SNPs. The red line indicates genome-wide significance level (5×10^−8^), and the horizontal lower line indicates suggestive evidence level (1 × 10^−5^).

A total of 1,338,796 eligible SNPs from 137 DZ twin pairs are included in bivariate GWA analysis. Q-Q plot (Fig 2) indicated no evidence of genomic inflation of test statistics or the bias from the population stratification. As illustrated in Manhattan plot (Fig 3), 27 SNPs of both BMI-SBP and BMI-DBP, and 25 SNPs of SBP-DBP reached suggestive evidence level (*P* < 1 × 10^−5^), although none of the SNPs exceeded statistically significant level (*P<*5×10^−8^).

Among top signals associated with BMI-SBP, the strongest associated SNP is rs200126670 (*P* = 2.94×10^−7^). Ten SNPs near *LINC00343* gene located on chromosome 13q33.2 (S2 Table). Among top signals associated with BMI-DBP, the strongest associated SNP is rs2025924 (*P* = 3.41×10^−7^). Eleven SNPs near *LINC00343* gene were located on chromosome 13q33.2. Two SNPs at *SLC39A11* gene located on chromosome 17q24.3-q25.1 (S3 Table). Among top signals associated with SBP-DBP, the strongest associated SNP is rs113511958 (*P* = 5.14×10^−7^). Three SNPs near *LINC00624* gene were located on chromosome 1q21.1-q21.2. And two SNPs at *TRAPPC9* gene were located on chromosome 8q24.3 (S4 Table). Moreover, rs4794029 is related with BMI-SBP, BMI-DBP, and SBP-DBP.

**Fig 4:**
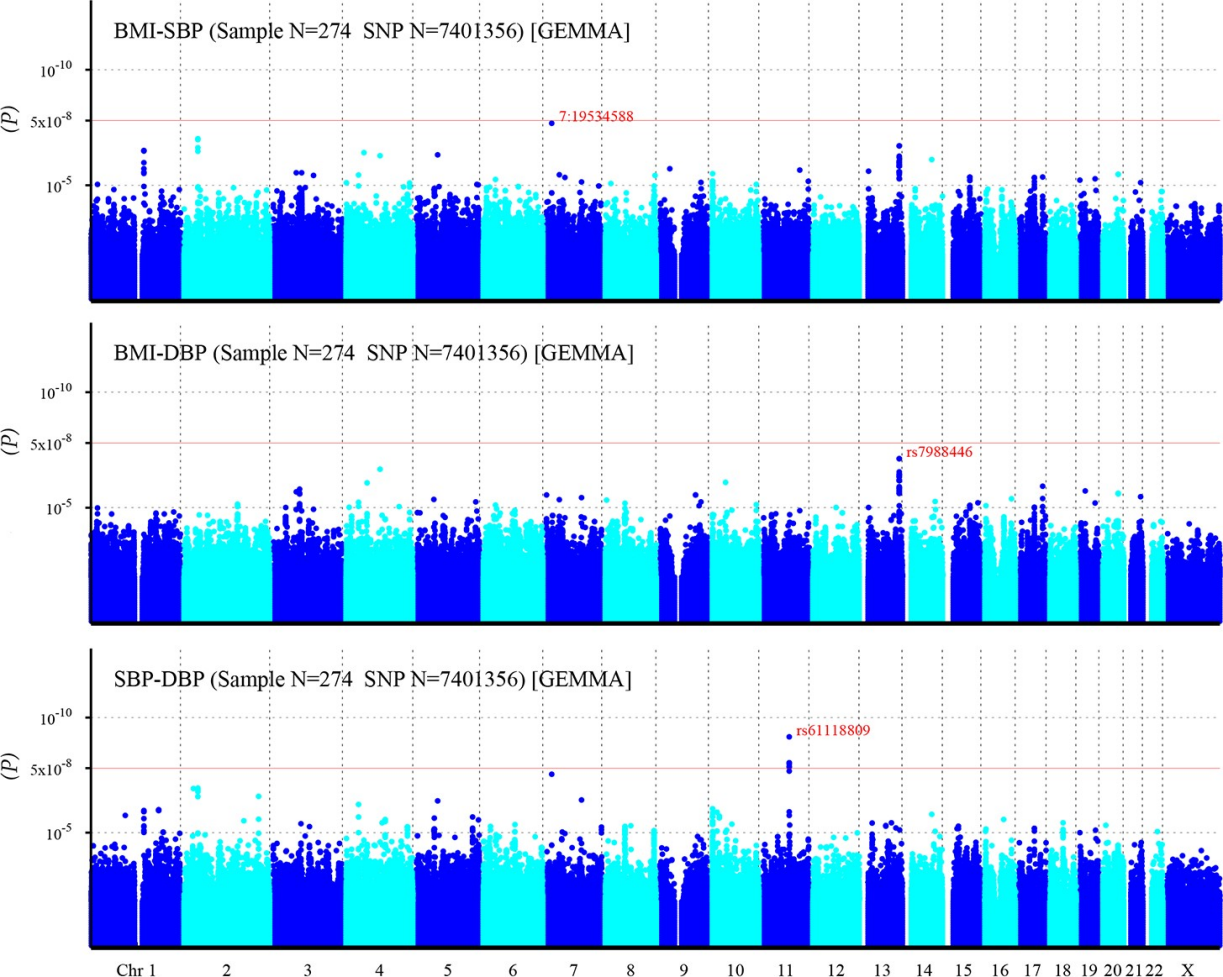
Manhattan plot of individual SNP associations with BMI-SBP, BMI-DBP, and SBP-DBP after imputation. For details see Fig 3.

After imputation, more risk variants are identified (Fig 4). One SNP related to BMI-SBP, and eight SNPs related to SBP-DBP reached significant statistic level. However, still no SNPs associated with BMI-DBP exceeds the significant threshold (Table 3).

**Table 3:** SNPs that reached *P* < 5×10^−8^ from bivariate GWAS of BMI-SBP, BMI-DBP and SBP-DBP after imputation.

SNP	Chr.	Position(bp)	*P*-value	Gene/Nearest gene
**BMI-SBP**				
7:19534588 (rs540063109)	7	19534588	2.06E-08	*TMEM196*
**SBP-DBP**				
rs61118809	11	78630891	6.84E-10	*TENM4*
rs5792827	11	78645913	9.14E-09	*TENM4*
rs5013388	11	78646117	9.14E-09	*TENM4*
rs79840843	11	78643557	1.04E-08	*TENM4*
rs57611205	11	78645171	1.34E-08	*TENM4*
rs673456	11	78629612	1.42E-08	*TENM4*
rs12419933	11	78630596	2.07E-08	*TENM4*
7:19534588 (rs540063109)	7	19534588	2.90E-08	TMEM196

Chr, chromosome. bp: base pair.

To eliminate the effects of linkage disequilibrium and identify independently associated variants, we re-evaluated each variant conditioning on the top variant, which located at chromosome 1, 13, and 5, respectively (S1–S3 Figs).

#### Expression quantitative trait loci (eQTL) analysis

We queried SNPs from S2–S4 Tables and Table 3 with LD threshold r^2^>0.8 and Asian population referenced. Among them, rs4794029 located on 17q21.32 was the significant eQTL in three tissues (Adipose-Subcutaneous (*P* = 8.4×10^−8^), Heart-Atrial Appendage (*P* = 1×10^−6^), and Heart-Left Ventricle (*P* = 2.5× 10^−5^)) related to BMI and SBP. As for SBP and DBP, we found significant eQTLs located on 17q21.32 and 8q12.1 that related to blood pressure, such as rs4400367, the significant eQTL in several tissues (Heart-Atrial Appendage (*P* = 2.7×10^−14^), and Heart-Left Ventricle(*P* = 7.3×10^−12^), Artery-Aorta(*P* = 1.2×10^−6^, *P* = 1.3×10^−5^), and Artery-Tibial (*P* = 1.7× 10^−5^)). However, none significant eQTL was found in tissues related to BMI and DBP (Table 4).

**Table 4:** *P*-values of SNPs in different tissues related to BMI-SBP, and SBP-DBP.

SNP ID	Tissue Site Detail	*P*-Value	Gene Symble
**BMI and SBP**			
rs4794029	Adipose—Subcutaneous	8.40E-08	*GNGT2*
	Heart—Atrial Appendage	1.00E-06	*GNGT2*
	Heart—Left Ventricle	2.50E-05	*GNGT2*
	Artery—Aorta	9.50E-05	*GNGT2*
**SBP and DBP**			
rs4400367	Heart—Atrial Appendage	2.70E-14	*SDCBP*
	Heart—Left Ventricle	7.30E-12	*SDCBP*
	Artery—Aorta	1.20E-06	*NSMAF*
	Artery—Aorta	1.30E-05	*SDCBP*
	Artery—Tibial	1.70E-05	*CYP7A1*
rs10113750	Heart—Atrial Appendage	6.90E-15	*SDCBP*
	Heart—Left Ventricle	2.00E-12	*SDCBP*
	Artery—Tibial	4.50E-06	*CYP7A1*
	Artery—Aorta	5.70E-06	*NSMAF*
	Artery—Aorta	1.30E-07	*SDCBP*
rs11776003	Heart—Atrial Appendage	1.40E-15	*SDCBP*
	Heart—Atrial Appendage	1.40E-15	*SDCBP*
	Heart—Left Ventricle	3.60E-12	*SDCBP*
	Artery—Aorta	1.40E-07	*SDCBP*
rs3739327	Heart—Atrial Appendage	3.30E-14	*SDCBP*
	Heart—Left Ventricle	2.10E-11	*SDCBP*
	Artery—Aorta	1.60E-06	*NSMAF*
	Artery—Aorta	1.40E-05	*SDCBP*
	Artery—Tibial	2.00E-05	*CYP7A1*
rs55978930	Heart—Atrial Appendage	2.90E-05	*GNGT2*
rs4794029	Heart—Atrial Appendage	1.00E-06	*GNGT2*
	Heart—Left Ventricle	2.50E-05	*GNGT2*
	Artery—Aorta	9.50E-05	*GNGT2*

#### Gene-based analysis

We found no genes exceeding the statistically significant level (2.63×10^−6^). However, 1043 genes of BMI-SBP, 1103 genes of BMI-DBP, and 1100 genes of SBP-DBP reached suggestive evidence level (*P* < 0.05). *PHOSPHO1* is related to both BMI-SBP and BMI-DBP with smallest *P-* value. Top 10 genes are displayed in Table 5 ordered by *P*- value.

**Table 5:** Top 10 genes that reached *P*<0.05 from bivariate GWAS of BMI-SBP, BMI-DBP and SBP-DBP.

Gene	Chr.	SNP(n)	From	To	*P*
**BMI-SBP**					
*PHOSPHO1*	17	2	47300731	47308128	6.00E-06
*GNGT2*	17	5	47283595	47287936	6.00E-06
*KEAP1*	19	5	10596795	10614054	6.40E-05
*NAP1L1*	12	11	76438671	76478738	1.53E-04
*ABI3*	17	8	47287588	47300587	1.65E-04
*PSMB3*	17	5	36908965	36920484	1.89E-04
*PFKFB3*	10	131	6186842	6277507	2.17E-04
*TP53I13*	17	2	27895738	27900175	2.34E-04
*S1PR5*	19	4	10623417	10628668	2.39E-04
*THRB*	3	317	24158644	24536313	2.75E-04
**BMI-DBP**					
*PHOSPHO1*	*17*	2	47300731	47308128	1.10E-05
*GNGT2*	17	5	47283595	47287936	2.40E-05
*LINC00346*	13	6	111516333	111522655	2.80E-05
*KEAP1*	19	5	10596795	10614054	6.20E-05
*FMO9P*	1	8	166573152	166594473	8.10E-05
*TFF2*	21	3	43766466	43771208	1.35E-04
*TP53I13*	17	2	27895738	27900175	1.75E-04
*FEV*	2	2	219845808	219850379	1.84E-04
*FBLIM1*	1	12	16085254	16113084	2.31E-04
*MRPS28*	8	28	80831094	80942506	2.62E-04
**SBP-DBP**					
*LINC00346*	13	6	111516333	111522655	4.00E-05
*TFF2*	21	3	43766466	43771208	9.20E-05
*SLC37A4*	11	7	118895060	118901616	2.00E-04
*TRAPPC4*	11	3	118889240	118894385	2.06E-04
*LOC339593*	20	12	11247306	11254031	2.18E-04
*LINC01158*	2	14	105421882	105467934	2.23E-04
*S100A9*	1	2	153330329	153333503	2.30E-04
*CLIP4*	2	45	29320541	29406679	2.32E-04
*TREML2*	6	14	41157486	41168925	2.37E-04
*REM2*	14	3	23352431	23356889	2.38E-04

Chr, chromosome.

In addition, above mentioned genes were queried in GTEx, and then number of significant eQTLs in interest tissues were displayed in S5 Table.

#### Pathway enrichment analysis

628 pathways were significantly associated with BMI-SBP, 599 with BMI-DBP, and 766 with SBP-DBP (*P* < 0.05), such as prion diseases, IL5 pathway, and Cyclin E associated events during G1/S transition, respectively. TGF beta signaling pathway, G βγ signaling through PI3Kγ, prolactin receptor signaling, ADP signaling through P2RY1 pathway etc. More details of top 20 pathways are displayed in S6 Table.

## Discussion

We conducted the first bivariate GWAS of joint BMI and BP phenotypes in northern Chinese population. This investigation included 380 twin pairs with mean age of 51.5±7.6 years; mean BMI of 24.3±3.3 kg/m^2^, and mean SBP and DBP of 130.8±20.1mmHg and 83.1±10.9mmHg, respectively. Bivariate structural equation models indicated a genetic component in phenotypic correlation between BMI and BP. The significant genetic correlation of BMI-SBP, BMI-DBP, and SBP-DBP were 0.2108, 0.2345, and 0.6942, respectively. Previous studies proved the genetic correlation between BMI and BP range from 0.15 to 0.49 [22, 56]. Therefore, the pleiotropic gene variants related to both BMI and BP need to be further explored.

This current GWAS identified 6 potential causal SNPs. rs4794029 may affect the gene expression level of *GNGT2* on BMI and SBP. GNGT2 was reported to be involved in inflammatory and immune responses through mediating β-arrestin 1-induced Akt phosphorylation and NFκB activation [57, 58]. We identified 6 SNPs in result of SBP and DBP. Of which, 3 eQTLs (rs4400367, rs10113750, rs3739327) effect *NSMAF* on gene expression level. NSMAF plays a pro-apoptotic role in tumor necrosis factor (TNF) delivering immune signals [59]. Also, lack of NSMAF result in a decrease in expression of interleukin 6 and various chemokines important in immunoregulation [60]. *CYP7A1* encodes a member of the cytochrome P450 superfamily of enzymes. Cytochrome (CYP) P450 metabolites of arachidonic acid contribute to the control of BP [61]. Previous studies confirmed other member of cytochrome P450 family such as *CYP17A1-CNNM2*, *CYP17A1*, *CYP1A2* genes associated with BP in Japanese and European population [25, 62].

In gene-based analysis, nine genes (*PHOSPHO1*, *GNGT2*, *KEAP1*, *S1PR5* etc.) were associated with both BMI-SBP and BMI-DBP. *PHOSPHO1* up-regulated express in rabbit models with hyperlipidemia and atherosclerosis [63]. Also, it has been proved that DNA methylation at *PHOSPHO1* in blood associated with type 2 diabetes risk [64, 65]. KEAP1 protein is one of key redox-sensitive signaling proteins mediating the response to oxidant stress, which inhibit regulator of transcription factor Nuclear factor-erythroid 2 p45-related factor 2 (Nrf2) [66]. The absence of Nrf2 could protect against weight gain and obesity [67]. *S1PR5* encodes one of receptors of sphingosine 1-phosphate (S1P), which activate the endothelial isoform of nitric-oxide synthase (eNOS) [68]. As a key signaling protein, eNOS promote relaxation of vascular smooth muscle and inhibit platelet aggregation [69].

In pathways enrichment analysis, 628 pathways were identified significant association with BMI-SBP, 599 pathways with BMI-DBP, and 766 pathways with SBP-DBP. Some of them were biologically plausible. G βγ signaling through PI3Kγ was found associated with BMI-DBP. The lipid kinase PI3Kγ, as a central proinflammatory signal transducer, proposed a role in obesity-induced inflammation and insulin resistance in hematopoietic cells [70, 71]. Previous research indicated the increasing PI3Kγ in insulin-resistant obese subjects. Meanwhile, PI3Kγ participants in physiological process of vascular smooth muscle cells [72]. Prolactin receptor signaling was found associated with SBP-DBP. Previous studies demonstrated that for each 5-mg/dL increment in prolactin, increased odds of low high-density lipoprotein cholesterol were observed in women. In men, a 5-mg/ dL increment was associated with increased risk of incident hypertension and diabetes [73].

To our best knowledge, this is the first GWAS about BMI and BP phenotypes focusing on northern Chinese population. Compared with univariate GWAS, bivariate design could identify more pleiotropic genes to reveal biological mechanisms related to obesity and hypertension. However, there are still some limitations existing. Firstly, the small twin sample due to difficulty in recruitment and restriction in inclusion criteria might be the obstacle to find more significant SNPs, genes and pathways. Secondly, due to lack of standard approach to define the cutoff *P* value in bivariate GWAS, 5 × 10^−8^ and 1 ×10^−5^, which based on Bonferroni correction, were still common used in current GWAS to solve multiple testing problem [74]. Since Bonferroni correction is quite conservative, we applied *P* <1 ×10^−5^ as the suggestive association in the discovery stage. However, low *P*-value threshold might lead to less significant genetic variants caught, thus, more further researches are required to explore the appropriate threshold. Thirdly, since different genetic background and sample size in limited researches, most of our result cannot be verified by other studies. Currently, many risk variants has been revealed associated with obesity and hypertension, however, most of them are lack of direct biological relationship with obesity and hypertension in pathophysiology.

In conclusion, present GWAS identified several SNPs, genes and pathways associated with both BMI and BP. Given there are still uncovered common genetic mechanism potentially related to obesity and hypertension, more studies need to be performed.

## Supporting information

S1 Table Full models and nested models of all traits. (DOCX)

S2 Table SNPs that reached *P* < 10–5 from bivariate GWAS of BMI-SBP. (DOCX)

S3 Table SNPs that reached *P* < 10–5 from bivariate GWAS of BMI-DBP. (DOCX)

S4 Table SNPs that reached *P* < 10–5 from bivariate GWAS of SBP-DBP. (DOCX)

S5 Table Numbers of significant eQTLs of top 10 genes in interest tissues related to BMI-SBP, BMI-DBP and SBP-DBP. (DOCX)

S6 Table The top 20 pathway results-KEGG, Reactome, and Biocarta (emp-*P* < 0.05) for BMI-SBP, BMI-DBP and SBP-DBP. (DOCX)

S1 Fig Regional association plot showing signal around chromosomal loci of 1q32.1 for bivariate genome-wide association study of BMI-SBP. (TIF)

S2 Fig Regional association plot showing signal around chromosomal loci of 13q33.2 for bivariate genome-wide association study of BMI-DBP. (TIF)

S3 Fig Regional association plot showing signal around chromosomal loci of 5q32 for bivariate genome-wide association study of SBP-DBP. (TIF)
