# Supplementary material for: Bivariate genome-wide association study (GWAS) of body mass index and blood pressure phenotypes in northern Chinese twins
Source: PLoS One. 2021 Feb 4;16(2):e0246436. doi: 10.1371/journal.pone.0246436 (PMC7861438; doi:10.1371/journal.pone.0246436)
Supplement: S1 Table — (DOCX) [file pone.0246436.s001.docx]

S1 Table. Full models and nested models of all traits.

| Traits | Models | A | D\|C | E | -2LL | df | AIC | Δχ2 | *Δdf* | *p* |
| --- | --- | --- | --- | --- | --- | --- | --- | --- | --- | --- |
| BMI | ADE | 66.4 (0.5-79.1) | 8.2 (0-74.3) | 25.5 (20.9-31.1) | 1929.7 | 754 | 421.7 |  |  |  |
|  | ACE | 74.5 (50.3-79.1) | 0 (0-23.3) | 25.5 (20.9-31.2) | 1929.8 | 754 | 421.8 |  |  |  |
|  | AE | 74.5 (68.9-79.1) | - | 25.5 (20.9-31.2) | 1929.8 | 755 | 419.8 | 0.1 | 1 | 0.79 |
|  | CE | - | 61.7 (55.1-67.6) | 38.3 (32.4-44.9) | 1971.0 | 755 | 461.0 | 41.3 | 1 | <0.001 |
| SBP | ADE | 53.5 (2.4-61.2) | 0 (0-51.9) | 46.5 (38.8-55.4) | 1946.8 | 754 | 438.8 |  |  |  |
|  | ACE | 45 (13-60.9) | 8 (0-36.3) | 47 (39-56.3) | 1946.5 | 754 | 438.5 |  |  |  |
|  | AE | 53.5 (44.6-61.2) | - | 46.5 (38.8-55.4) | 1946.8 | 755 | 436.8 | 0.0 | 1 | 1.00 |
|  | CE | - | 45.1 (36.7-52.8) | 54.9 (47.2-63.3) | 1954.4 | 755 | 444.4 | 7.6 | 1 | 0.01 |
| DBP | ADE | 48.9 (2.4-57.1) | 0 (0-47.3) | 51.1 (42.9-60.4) | 2003.8 | 754 | 495.8 |  |  |  |
|  | ACE | 35.6 (2.5-56.5) | 12.6 (0-41.5) | 51.9 (43.3-61.7) | 2003.2 | 754 | 495.2 |  |  |  |
|  | AE | 48.9 (39.6-57.1) | - | 51.1 (42.9-60.4) | 2003.8 | 755 | 493.8 | 0.0 | 1 | 1.00 |
|  | CE | - | 42.1 (33.4-50.1) | 57.9 (49.9-66.6) | 2007.7 | 755 | 497.7 | 3.9 | 1 | 0.05 |

A, D|C, and E : proportion of additive genetic, dominant genetic|shared environmental, and unique environmental variance of every indicator; AIC: Akaike’s information criterion.
