# Supplementary material for: Bivariate genome-wide association study (GWAS) of body mass index and blood pressure phenotypes in northern Chinese twins
Source: PLoS One. 2021 Feb 4;16(2):e0246436. doi: 10.1371/journal.pone.0246436 (PMC7861438; doi:10.1371/journal.pone.0246436)
Supplement: S2 Table — (DOCX) [file pone.0246436.s002.docx]

S2 Table. SNPs that reached *P* < 10^-5^ from bivariate GWAS of BMI-SBP.

| **SNP** | **Chr.** | **Position(bp)** | ***P-*value** | **Gene/Nearest gene**  **gene gene** |
| --- | --- | --- | --- | --- |
| 1:201008796(rs200126670) | 1 | 201008796 | 2.94E-07 | *KIF21B* |
| rs34710727 | 1 | 146997592 | 3.31E-07 | *LINC00624* |
| rs2025924 | 13 | 106446477 | 6.29E-07 | *LINC00343* |
| rs78826453 | 13 | 106451587 | 1.04E-06 | *LINC00343* |
| rs80230511 | 13 | 106466262 | 1.04E-06 | *LINC00343* |
| rs34284694 | 1 | 146996801 | 1.08E-06 | *LINC00624* |
| rs729426 | 13 | 106480081 | 1.44E-06 | *LINC00343* |
| rs13378734 | 13 | 106485240 | 1.44E-06 | *LINC00343* |
| rs35465657 | 1 | 146994436 | 1.96E-06 | *LINC00624* |
| rs62408297 | 6 | 70162205 | 2.24E-06 | *ADGRB3* |
| rs7335212 | 13 | 106476076 | 2.52E-06 | *LINC00343* |
| rs72663838 | 13 | 106487254 | 2.53E-06 | *LINC00343* |
| rs59412652 | 13 | 106488121 | 2.53E-06 | *LINC00343* |
| rs9586994 | 13 | 106488608 | 2.53E-06 | *LINC00343* |
| rs12912024 | 15 | 70585932 | 4.42E-06 | *LOC105370878* |
| rs617182 | 17 | 47307274 | 4.51E-06 | *PHOSPHO1* |
| rs4794029 | 17 | 47280301 | 4.75E-06 | *GNGT2* |
| rs79817709 | 19 | 10596872 | 4.76E-06 | *KEAP1* |
| rs7987002 | 13 | 106447755 | 5.06E-06 | *LINC00343* |
| rs57143316 | 6 | 39069291 | 5.49E-06 | *SAYSD1* |
| rs73451216 | 7 | 134242492 | 5.66E-06 | *AKR1B15* |
| rs1529767 | 15 | 70585318 | 6.15E-06 | *LOC105370878* |
| rs2501306 | 1 | 22342837 | 6.78E-06 | *CELA3A* |
| rs55978930 | 17 | 47299789 | 6.92E-06 | *ABI3* |
| rs10853110 | 17 | 47318414 | 7.05E-06 | *FLJ40194* |
| rs76039363 | 3 | 127292100 | 8.39E-06 | *TPRA1* |
| rs812619 | 5 | 173212571 | 9.01E-06 | *LOC107986482* |

Chr, chromosome. bp: base pair.
