# Supplementary material for: Bivariate genome-wide association study (GWAS) of body mass index and blood pressure phenotypes in northern Chinese twins
Source: PLoS One. 2021 Feb 4;16(2):e0246436. doi: 10.1371/journal.pone.0246436 (PMC7861438; doi:10.1371/journal.pone.0246436)
Supplement: S3 Table — (DOCX) [file pone.0246436.s003.docx]

S3 Table. SNPs that reached *P* < 10^-5^ from bivariate GWAS of BMI-DBP.

| **SNP** | **Chr.** | **Position(bp)** | ***P-*value** | **Gene/Nearest gene** |
| --- | --- | --- | --- | --- |
| rs2025924 | 13 | 106446477 | 3.41E-07 | *LINC00343* |
| rs78826453 | 13 | 106451587 | 4.46E-07 | *LINC00343* |
| rs80230511 | 13 | 106466262 | 4.46E-07 | *LINC00343* |
| rs729426 | 13 | 106480081 | 6.35E-07 | *LINC00343* |
| rs13378734 | 13 | 106485240 | 6.35E-07 | *LINC00343* |
| rs72663838 | 13 | 106487254 | 1.36E-06 | *LINC00343* |
| rs59412652 | 13 | 106488121 | 1.36E-06 | *LINC00343* |
| rs9586994 | 13 | 106488608 | 1.36E-06 | *LINC00343* |
| rs7335212 | 13 | 106476076 | 1.76E-06 | *LINC00343* |
| rs78992800 | 13 | 38939829 | 1.98E-06 | *UFM1* |
| rs7987002 | 13 | 106447755 | 2.38E-06 | *LINC00343* |
| rs1507413 | 3 | 78610831 | 2.49E-06 | *ROBO1* |
| rs72902721 | 3 | 78613444 | 2.49E-06 | *ROBO1* |
| rs3924958 | 17 | 70645446 | 3.97E-06 | *SLC39A11* |
| rs79817709 | 19 | 10596872 | 4.61E-06 | *KEAP1* |
| rs144107579 | 23 | 44022877 | 4.70E-06 | *EFHC2* |
| rs3804250 | 5 | 169722897 | 5.44E-06 | *LCP2* |
| rs9682532 | 3 | 78609963 | 6.67E-06 | *MRPS17P3* |
| rs702640 | 5 | 53256679 | 6.98E-06 | *ARL15* |
| rs57143316 | 6 | 39069291 | 7.49E-06 | *SAYSD1* |
| rs12912024 | 15 | 70585932 | 7.91E-06 | *LOC105370878* |
| rs7320405 | 13 | 106482474 | 8.00E-06 | *LINC00343* |
| 1:201008796(rs200126670) | 1 | 201008796 | 8.41E-06 | *KIF21B* |
| rs4312352 | 17 | 70650510 | 8.93E-06 | *SLC39A11* |
| rs13083642 | 3 | 78608901 | 8.96E-06 | *MRPS17P3* |
| rs4794029 | 17 | 47280301 | 9.50E-06 | *GNGT2* |
| rs617182 | 17 | 47307274 | 9.96E-06 | *PHOSPHO1* |

Chr, chromosome. bp: base pair.
