# Supplementary material for: Bivariate genome-wide association study (GWAS) of body mass index and blood pressure phenotypes in northern Chinese twins
Source: PLoS One. 2021 Feb 4;16(2):e0246436. doi: 10.1371/journal.pone.0246436 (PMC7861438; doi:10.1371/journal.pone.0246436)
Supplement: S4 Table — (DOCX) [file pone.0246436.s004.docx]

S4 Table. SNPs that reached *P* < 10^-5^ from bivariate GWAS of SBP-DBP.

| **SNP** | **Chr.** | **Position(bp)** | ***P-*value** | **Gene/Nearest gene** |
| --- | --- | --- | --- | --- |
| rs113511958 | 5 | 147208036 | 5.14E-07 | *SPINK1* |
| rs34710727 | 1 | 146997592 | 1.11E-06 | *LINC00624* |
| rs504245 | 11 | 78625883 | 1.77E-06 | *TENM4* |
| rs34284694 | 1 | 146996801 | 2.52E-06 | *LINC00624* |
| rs29413 | 4 | 114186005 | 3.23E-06 | *ANK2* |
| rs9875783 | 3 | 82477805 | 4.11E-06 | *LINC02008* |
| rs2332838 | 4 | 175526690 | 5.73E-06 | *LOC105377549* |
| rs3739327 | 8 | 59496052 | 6.33E-06 | *NSMAF* |
| rs4794029 | 17 | 47280301 | 6.51E-06 | *GNGT2* |
| rs1023674 | 5 | 54265532 | 6.95E-06 | *ESM1* |
| rs4400367 | 8 | 59489549 | 7.34E-06 | *SDCBP* |
| rs17107001 | 10 | 89686509 | 7.64E-06 | *PTEN* |
| rs1426938 | 4 | 175500748 | 7.68E-06 | *LOC105377549* |
| rs67701708 | 8 | 140916796 | 7.95E-06 | *TRAPPC9* |
| rs1075493 | 8 | 140917457 | 7.95E-06 | *TRAPPC9* |
| rs35465657 | 1 | 146994436 | 8.19E-06 | *LINC00624* |
| rs11776003 | 8 | 59459972 | 8.42E-06 | *SDCBP* |
| rs10113750 | 8 | 59464692 | 8.42E-06 | *SDCBP* |
| rs72815554 | 5 | 160995760 | 8.46E-06 | *GABRB2* |
| rs55978930 | 17 | 47299789 | 8.46E-06 | *ABI3* |
| rs4129044 | 16 | 6901513 | 8.73E-06 | *RBFOX1* |
| rs11256258 | 10 | 6033415 | 8.81E-06 | *IL 15RA* |
| rs8095594 | 18 | 41022827 | 9.07E-06 | *SYT4* |
| rs34666749 | 18 | 41025786 | 9.07E-06 | *SYT4* |

Chr, chromosome. bp: base pair.
