# Supplementary material for: Bivariate genome-wide association study (GWAS) of body mass index and blood pressure phenotypes in northern Chinese twins
Source: PLoS One. 2021 Feb 4;16(2):e0246436. doi: 10.1371/journal.pone.0246436 (PMC7861438; doi:10.1371/journal.pone.0246436)
Supplement: S5 Table — (DOCX) [file pone.0246436.s005.docx]

S5 Table. Numbers of significant eQTLs of top 10 genes in interest tissues related to BMI-SBP, BMI-DBP and SBP-DBP.

| **Top 20 gene** | **Adipose-Subcutaneous** | **Adipose-Visceral (Omentum)** | **Artery - Aorta** | **Artery - Coronary** | **Artery - Tibial** | **Brain-cerebellar-hemisphere** | **Heart - Atrial Appendage** | **Heart - Left Ventricle** |
| --- | --- | --- | --- | --- | --- | --- | --- | --- |
| **BMI and SBP** |  |  |  |  |  |  |  |  |
| *PHOSPHO1* | none | 8 | none | none | none | none | none | 1 |
| *GNGT2* | 109 | 12 | 18 | none | 2 | none | 6 | 10 |
| *KEAP1* | none | 73 | 25 | none | none | 11 | 3 | 30 |
| *NAP1L1* | none | none | 5 | none | 89 | none | 4 | none |
| *ABI3* | none | 2 | none | none | none | none | none | none |
| *PSMB3* | 76 | 38 | 93 | 13 | 104 | none | 30 | 38 |
| *PFKFB3* | 1 | none | none | none | none | none | none | 24 |
| *TP53I13* | 38 | 21 | 189 | 13 | 25 | none | 238 | 117 |
| *S1PR5* | none | none | 3 | none | 23 | 2 | none | none |
| *THRB* | 16 | none | 24 | none | 43 | none | 81 | 28 |
| **BMI and DBP** |  |  |  |  |  |  |  |  |
| *PHOSPHO1* | none | 8 | none | none | none | none | none | 1 |
| *GNGT2* | 109 | 12 | 18 | none | 2 | none | 6 | 10 |
| *LINC00346* | 43 | 4 | 18 | 4 | 111 | 5 | 32 | 18 |
| *KEAP1* | none | 73 | 25 | none | none | 11 | 3 | 30 |
| *FMO9P* | none | none | none | none | none | none | none | none |
| *TFF2* | none | none | none | none | none | none | none | none |
| *TP53I13* | 38 | 21 | 189 | 13 | 25 | none | 238 | 117 |
| *FEV* | none | none | none | none | none | none | none | none |
| *FBLIM1* | 58 | none | 46 | 21 | 73 | none | none | none |
| *MRPS28* | none | none | none | none | none | none | none | none |
| **SBP and DBP** |  |  |  |  |  |  |  |  |
| *LINC00346* | 43 | 4 | 18 | 4 | 111 | 5 | 32 | 18 |
| *TFF2* | none | none | none | none | none | none | none | none |
| *SLC37A4* | 126 | 135 | 144 | 34 | 175 | none | 74 | 11 |
| *TRAPPC4* | 255 | 219 | 215 | 172 | 409 | 130 | 196 | 170 |
| *LOC339593* | none | none | none | none | none | none | none | none |
| *LINC01158* | 11 | 123 | none | none | none | 43 | none | none |
| *S100A9* | 104 | 75 | 95 | none | 3 | 2 | none | 100 |
| *CLIP4* | 44 | none | none | none | 66 | none | 4 | none |
| *TREML2* | 3 | none | none | none | none | none | none | none |
| *REM2* | 2 | 5 | 14 | 1 | none | none | none | none |
