# Supplementary material for: Bivariate genome-wide association study (GWAS) of body mass index and blood pressure phenotypes in northern Chinese twins
Source: PLoS One. 2021 Feb 4;16(2):e0246436. doi: 10.1371/journal.pone.0246436 (PMC7861438; doi:10.1371/journal.pone.0246436)
Supplement: S6 Table — (DOCX) [file pone.0246436.s006.docx]

S6 Table. The top 20 pathway results-KEGG, Reactome, and Biocarta (emp-*P* < 0.05) for BMI-SBP, BMI-DBP and SBP-DBP.

| **Pathway** | **emp-*P*** | **-log(emp-*P*)** |
| --- | --- | --- |
| **BMI-SBP** |  |  |
| KEGG_PRION_DISEASES | 1.48E-04 | 3.83 |
| REACTOME_CELL_JUNCTION_ORGANIZATION | 3.30E-04 | 3.48 |
| REACTOME_SYNTHESIS_OF_PC | 3.94E-04 | 3.40 |
| REACTOME_SIGNALING_BY_ERBB4 | 5.12E-04 | 3.29 |
| BIOCARTA_EGFR_SMRTE_PATHWAY | 6.50E-04 | 3.19 |
| REACTOME_CELL_CELL_COMMUNICATION | 8.10E-04 | 3.09 |
| REACTOME_NUCLEAR_RECEPTOR_TRANSCRIPTION_PATHWAY | 1.03E-03 | 2.99 |
| BIOCARTA_MAL_PATHWAY | 1.12E-03 | 2.95 |
| BIOCARTA_CDK5_PATHWAY | 1.15E-03 | 2.94 |
| REACTOME_GRB2_EVENTS_IN_ERBB2_SIGNALING | 1.15E-03 | 2.94 |
| BIOCARTA_BARR_MAPK_PATHWAY | 1.16E-03 | 2.94 |
| REACTOME_G_ALPHA_Z_SIGNALLING_EVENTS | 1.17E-03 | 2.93 |
| REACTOME_SOS_MEDIATED_SIGNALLING | 1.17E-03 | 2.93 |
| KEGG_SPLICEOSOME | 1.19E-03 | 2.92 |
| REACTOME_RAF_MAP_KINASE_CASCADE | 1.24E-03 | 2.91 |
| REACTOME_SIGNALING_BY_BMP | 1.25E-03 | 2.90 |
| BIOCARTA_HCMV_PATHWAY | 1.28E-03 | 2.89 |
| REACTOME_SHC1_EVENTS_IN_ERBB4_SIGNALING | 1.29E-03 | 2.89 |
| BIOCARTA_ALK_PATHWAY | 1.30E-03 | 2.89 |
| REACTOME_SIGNALLING_TO_P38_VIA_RIT_AND_RIN | 1.33E-03 | 2.88 |
| **BMI-DBP** |  |  |
| BIOCARTA_IL5_PATHWAY | 2.79E-04 | 3.55 |
| REACTOME_ADP_SIGNALLING_THROUGH_P2RY12 | 7.40E-04 | 3.13 |
| REACTOME_INHIBITION_OF_INSULIN_SECRETION_BY_ADRENALINE_NORADRENALINE | 7.60E-04 | 3.12 |
| REACTOME_ADP_SIGNALLING_THROUGH_P2RY1 | 7.60E-04 | 3.12 |
| BIOCARTA_KERATINOCYTE_PATHWAY | 7.90E-04 | 3.10 |
| REACTOME_G_BETA_GAMMA_SIGNALLING_THROUGH_PLC_BETA | 8.00E-04 | 3.10 |
| REACTOME_G_PROTEIN_ACTIVATION | 8.10E-04 | 3.09 |
| REACTOME_ACTIVATION_OF_KAINATE_RECEPTORS_UPON_GLUTAMATE_BINDING | 8.10E-04 | 3.09 |
| REACTOME_PROSTACYCLIN_SIGNALLING_THROUGH_PROSTACYCLIN_RECEPTOR | 8.40E-04 | 3.08 |
| KEGG_WNT_SIGNALING_PATHWAY | 1.02E-03 | 2.99 |
| REACTOME_SYNTHESIS_OF_PC | 1.19E-03 | 2.92 |
| KEGG_ASTHMA | 1.26E-03 | 2.90 |
| REACTOME_G_BETA_GAMMA_SIGNALLING_THROUGH_PI3KGAMMA | 1.44E-03 | 2.84 |
| KEGG_TGF_BETA_SIGNALING_PATHWAY | 1.45E-03 | 2.84 |
| REACTOME_SYNTHESIS_OF_PE | 1.48E-03 | 2.83 |
| REACTOME_G_PROTEIN_BETA_GAMMA_SIGNALLING | 1.60E-03 | 2.80 |
| REACTOME_G_ALPHA_Z_SIGNALLING_EVENTS | 1.79E-03 | 2.75 |
| REACTOME_CELL_EXTRACELLULAR_MATRIX_INTERACTIONS | 1.82E-03 | 2.74 |
| REACTOME_CYCLIN_A_B1_ASSOCIATED_EVENTS_DURING_G2_M_TRANSITION | 1.91E-03 | 2.72 |
| REACTOME_THROMBOXANE_SIGNALLING_THROUGH_TP_RECEPTOR | 2.00E-03 | 2.70 |
| **SBP-DBP** |  |  |
| REACTOME_CYCLIN_E_ASSOCIATED_EVENTS_DURING_G1_S_TRANSITION_ | 3.01E-05 | 4.52 |
| REACTOME_SIGNALING_BY_WNT | 3.18E-05 | 4.50 |
| REACTOME_INTEGRIN_CELL_SURFACE_INTERACTIONS | 3.22E-05 | 4.49 |
| REACTOME_REGULATION_OF_MITOTIC_CELL_CYCLE | 3.24E-05 | 4.49 |
| REACTOME_SCFSKP2_MEDIATED_DEGRADATION_OF_P27_P21 | 3.31E-05 | 4.48 |
| REACTOME_SCF_BETA_TRCP_MEDIATED_DEGRADATION_OF_EMI1 | 3.42E-05 | 4.47 |
| REACTOME_SIGNALING_BY_ERBB4 | 5.50E-05 | 4.26 |
| REACTOME_ACTIVATION_OF_NF_KAPPAB_IN_B_CELLS | 7.20E-05 | 4.14 |
| REACTOME_SIGNALING_BY_THE_B_CELL_RECEPTOR_BCR | 7.30E-05 | 4.14 |
| REACTOME_DOWNSTREAM_SIGNALING_EVENTS_OF_B_CELL_RECEPTOR_BCR | 8.60E-05 | 4.07 |
| REACTOME_ADAPTIVE_IMMUNE_SYSTEM | 2.94E-04 | 3.53 |
| KEGG_NEUROACTIVE_LIGAND_RECEPTOR_INTERACTION | 5.67E-04 | 3.25 |
| REACTOME_ANTIGEN_PROCESSING_UBIQUITINATION_PROTEASOME_DEGRADATION | 5.80E-04 | 3.24 |
| BIOCARTA_EGFR_SMRTE_PATHWAY | 7.60E-04 | 3.12 |
| REACTOME_CLASS_I_MHC_MEDIATED_ANTIGEN_PROCESSING_PRESENTATION | 7.70E-04 | 3.11 |
| REACTOME_PROLACTIN_RECEPTOR_SIGNALING | 7.90E-04 | 3.10 |
| REACTOME_NUCLEAR_SIGNALING_BY_ERBB4 | 8.70E-04 | 3.06 |
| BIOCARTA_SKP2E2F_PATHWAY | 9.00E-04 | 3.05 |
| REACTOME_NUCLEAR_RECEPTOR_TRANSCRIPTION_PATHWAY | 1.08E-03 | 2.97 |
| BIOCARTA_P27_PATHWAY | 1.18E-03 | 2.93 |

emp-*P*, empirical *p*-value.
