# Supplementary figures and images for: Bivariate genome-wide association study (GWAS) of body mass index and blood pressure phenotypes in northern Chinese twins
Source: PLoS One. 2021 Feb 4;16(2):e0246436. doi: 10.1371/journal.pone.0246436 (PMC7861438; doi:10.1371/journal.pone.0246436)

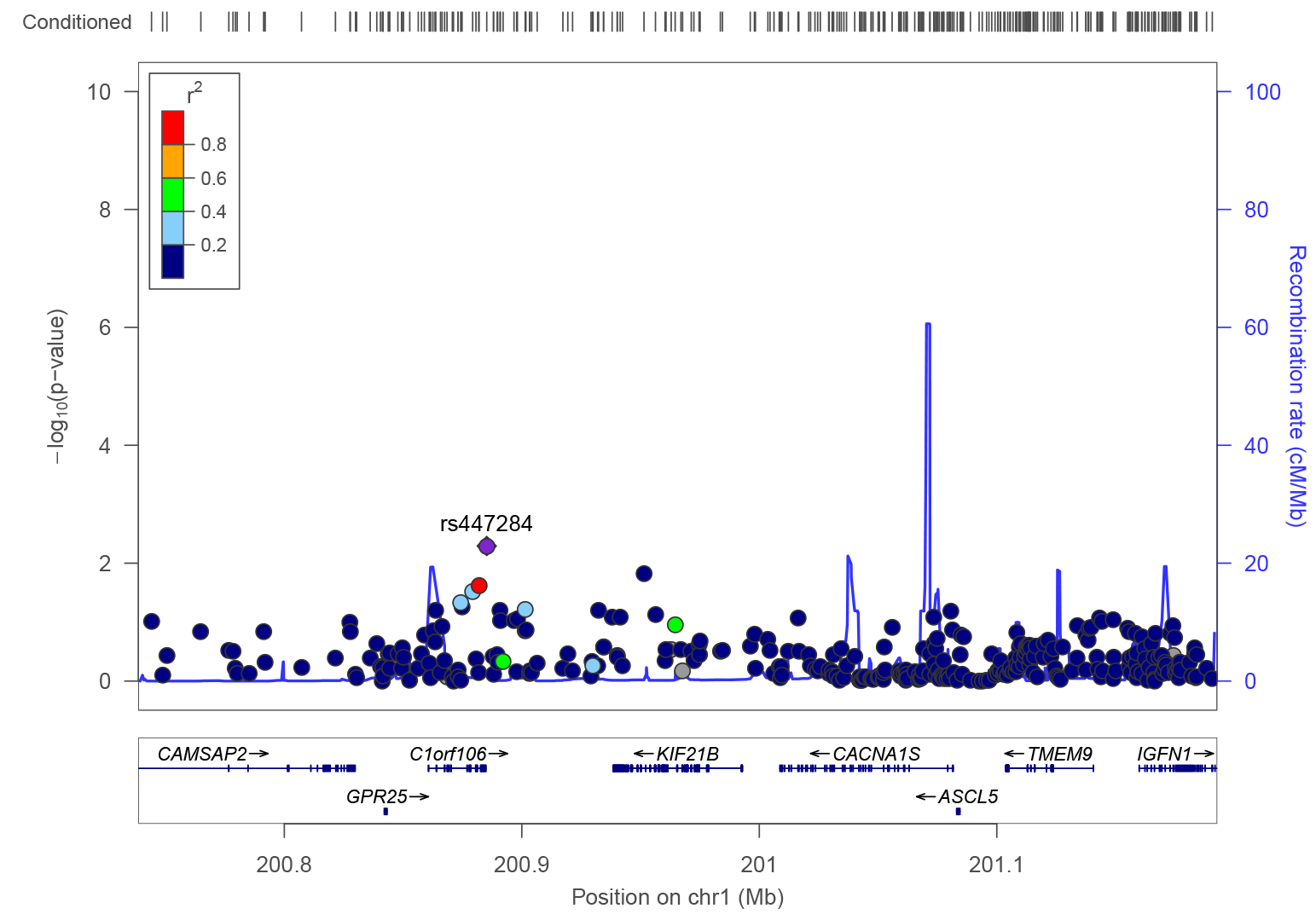

Supplement: S1 Fig — (TIF) [file pone.0246436.s007.tif]

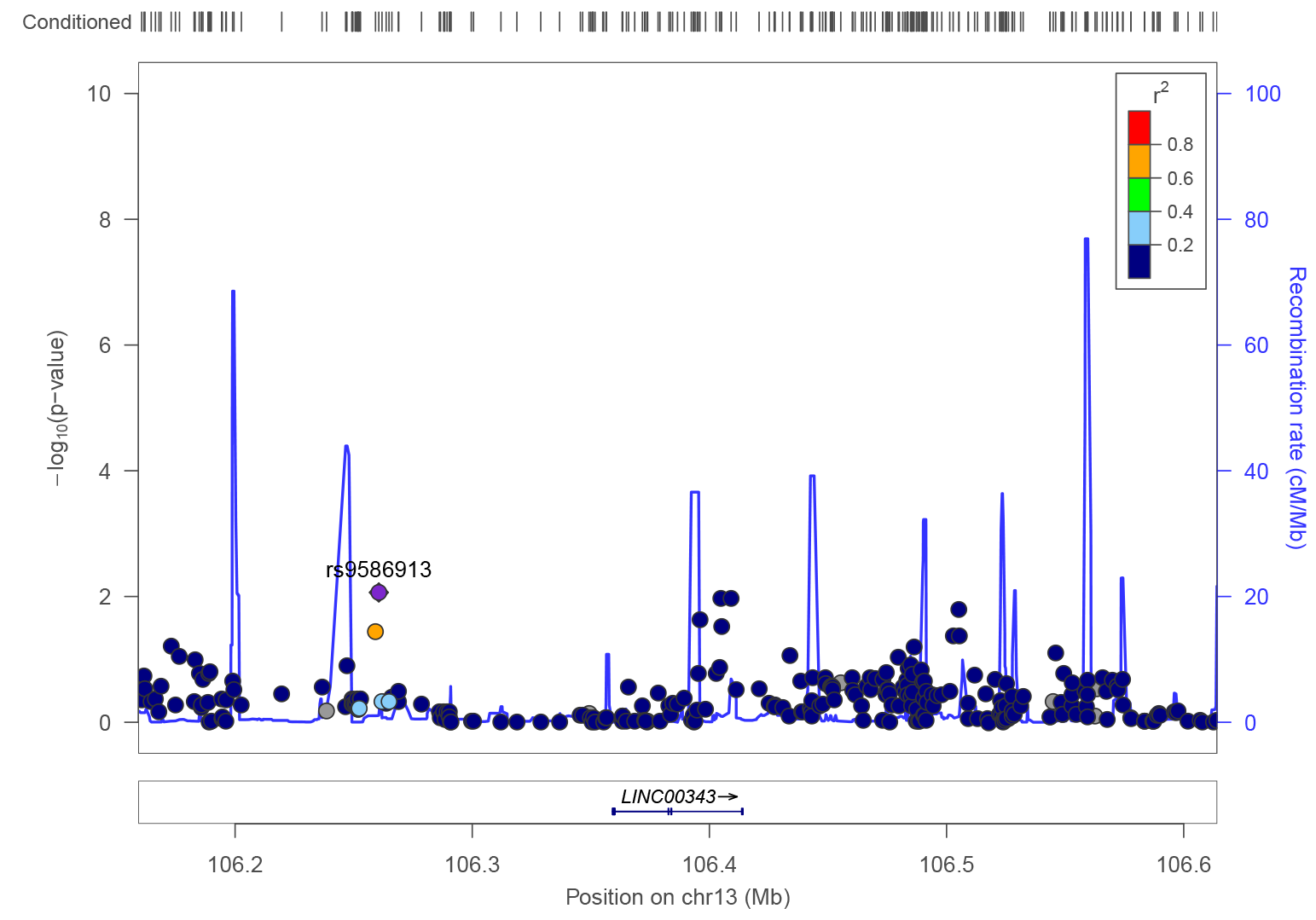

Supplement: S2 Fig — (TIF) [file pone.0246436.s008.tif]

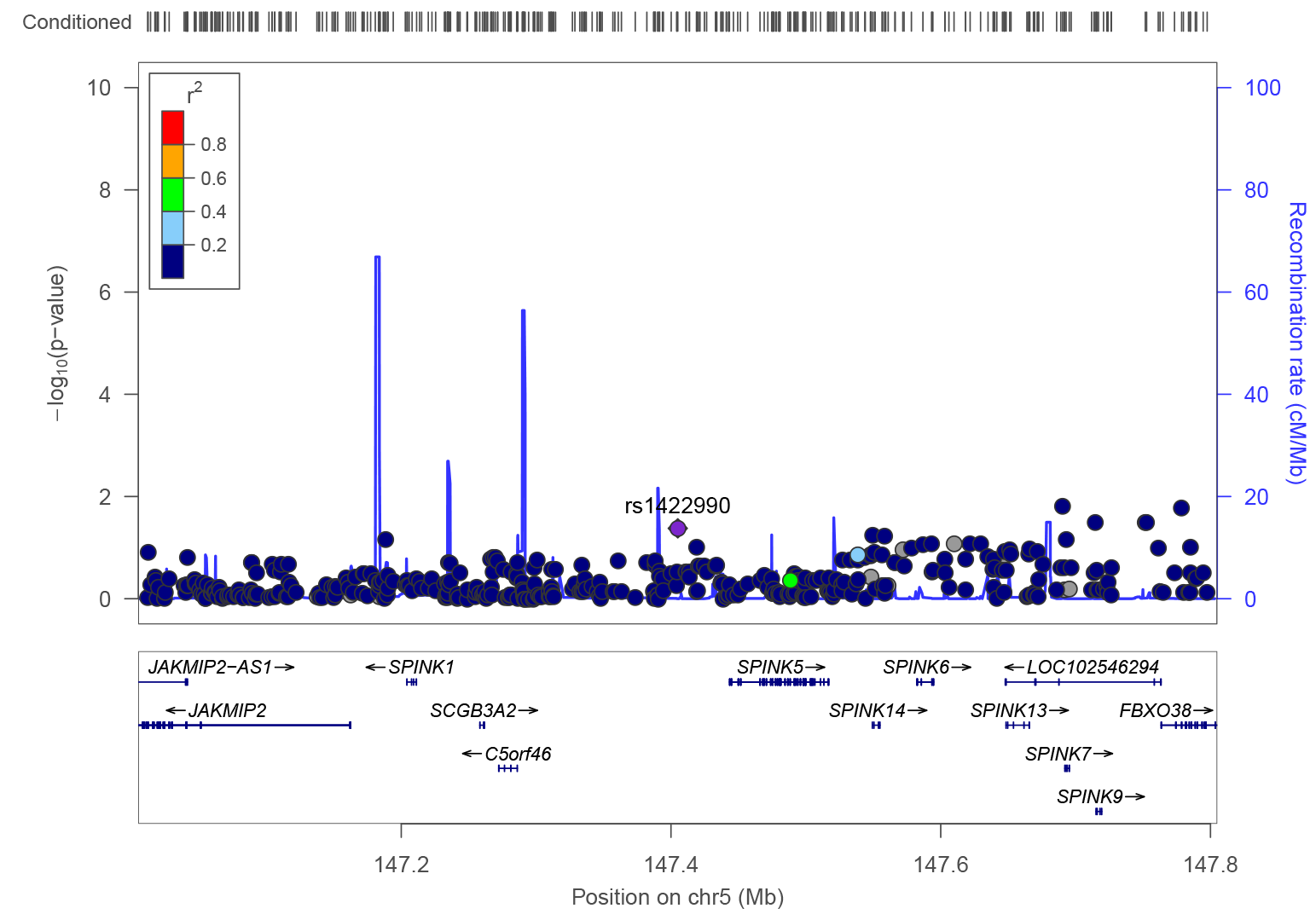

Supplement: S3 Fig — (TIF) [file pone.0246436.s009.tif]
